# Evaluation of a COX-2 fluorescent probe for adjunctive quantitative assessment of BV-associated inflammatory severity

**DOI:** 10.3389/fmicb.2026.1900748

**Published:** 2026-08-28

**Authors:** Yanlin Chen, Liying Wang, Tianjing Chen, Tianni You, Heng Xue, Yuanjun Cai, Jun Sheng, Lijun Xie, Lifang Xue, Zengzi Zhou, Liangzhi Cai

**Affiliations:** 1 College of Clinical Medicine for Obstetrics & Gynecology and Pediatrics, Fujian Medical University, Fuzhou, Fujian, China; 2 Laboratory of Gynecologic Oncology, Fujian Maternity and Child Health Hospital, College of Clinical Medicine for Obstetrics & Gynecology and Pediatrics, Fujian Medical University, Fuzhou, Fujian, China; 3 Fujian Key Laboratory of Women and Children's Critical Diseases Research, Fujian Maternity and Child Health Hospital (Fujian Women and Children's Hospital), Fuzhou, Fujian, China; 4 Fujian Clinical Research Center for Gynecological Oncology, Fujian Maternity and Child Health Hospital (Fujian Obstetrics and Gynecology Hospital), Fuzhou, Fujian, China; 5 Department of Laboratory Medicine, Fujian Maternity and Child Health Hospital, Fuzhou, Fujian, China; 6 Fujian Provincial Cervical Disease Diagnosis and Treatment Health Center, Fujian Maternity and Child Health Hospital, Fuzhou, Fujian, China; 7 Fujian Provincial Key Laboratory of Screening for Novel Microbial Products, Fujian Institute of Microbiology, Fuzhou, Fujian, China

**Keywords:** adjunctive diagnostic biomarker, bacterial vaginosis, COX-2 fluorescent probe, disease severity, vaginal inflammation

## Abstract

**Background and objective:**

Bacterial vaginosis (BV) is a prevalent lower genital tract infection linked to significant reproductive health complications. Current diagnostics are subjective or non-quantitative for clinical use. This study aims to evaluate a novel cyclooxygenase-2 (COX-2)-targeting fluorescent probe as an adjunctive quantitative tool for assessing BV-associated inflammatory severity within a standard BV diagnostic workflow.

**Methods:**

A murine BV model was established using estrogen-pretreated C57BL/6J mice infected vaginally with pathogens. Vaginal lavage samples from both BV and sham control mice were analyzed with the COX-2 probe. Clinically, vaginal secretions from 45 outpatients were grouped into Normal and BV categories based on microecological criteria. COX-2 fluorescence was measured in all samples and correlated with microbiological parameters.

**Results:**

The BV model showed elevated IL-1β and reduced IL-10 (*P* < 0.05), with significantly higher COX-2 fluorescence in BV mice (*P* < 0.05). Clinically, the BV group exhibited stronger COX-2 signals than the normal group (*P* < 0.05). Fluorescence intensity correlated with vaginal cleanliness, Nugent score, microbiota type, clue cells, and bacterial density (all *P* < 0.05).

**Conclusions:**

The COX-2-targeting nano-probe demonstrates strong correlation with key BV indicators and enables quantitative severity assessment. It represents a promising adjunctive quantitative tool for objective and stratified assessment of BV-associated inflammatory severity, with potential for clinical translation.

## 1 Background

Bacterial vaginosis (BV) is a prevalent lower genital tract infection characterized by vaginal dysbiosis (Abou Chacra et al., 2021; Zhang et al., 2022). It has a high global prevalence and is significantly associated with pelvic inflammatory disease, infertility, increased risk of sexually transmitted infections, and adverse pregnancy outcomes (McKinnon et al., 2019; Ravel et al., 2021). Despite its high incidence, approximately 10%−40% of affected individuals are asymptomatic (Coudray and Madhivanan, 2020), increasing the risk of missed diagnosis and underscoring the need for rapid and accurate diagnostic methods.

Current clinical diagnosis primarily relies on Amsel's criteria and Nugent scoring (Redelinghuys et al., 2020). However, Amsel's criteria are susceptible to subjectivity and are time-consuming (Coleman and Gaydos, 2018). The Nugent score, considered the diagnostic gold standard, also faces limitations, including a lack of standardized protocols and reduced diagnostic accuracy in specific populations, such as postmenopausal women (Mitchell et al., 2021). Notably, neither method enables objective, quantitative assessment of BV-associated inflammatory severity, which is closely linked to adverse reproductive outcomes. A fluorescence-based COX-2 probe could help address this gap by providing an objective, quantitative readout of BV-associated inflammatory severity.

Cyclooxygenase-2 (COX-2) is a key inducible enzyme in inflammatory responses and is markedly upregulated during bacterial infections. Studies have shown increased COX-2 expression in vaginal tissues of mice infected with *Gardnerella vaginalis* (GV), suggesting its involvement in BV-associated inflammation (Kwon et al., 2024; Li et al., 2023; Joo et al., 2011). Thus, we hypothesize that COX-2 can serve as a quantitative marker of BV-associated inflammatory severity, providing adjunctive diagnostic value within a standardized BV diagnostic workflow. Nevertheless, direct evidence regarding COX-2 expression patterns in vaginal secretions of human patients with BV, its correlation with inflammatory severity, and its potential utility as a quantitative biomarker for BV severity grading remains scarce.

Addressing these diagnostic challenges, visualization of COX-2 as a biomarker holds promise. A novel COX-2 fluorescent probe, compound 2a1, was previously developed by the Fujian Institute of Microbiology through structural optimization of rofecoxib. Its chemical structure, synthesis, selectivity, and photophysical properties have been fully characterized in a previous study (Xie et al., 2021).

Given the limitations of existing BV diagnostic methods and the potential role of COX-2 in BV-related inflammation, this study aimed to evaluate the feasibility of utilizing the novel COX-2 fluorescent probe (2a1) for quantitative assessment of BV-associated inflammatory severity. We focused on assessing its capability for visual detection of COX-2 expression in vaginal secretions, with the goal of achieving objective quantification of BV lesion severity. This approach could provide a simple and cost-effective auxiliary diagnostic strategy for clinical practice.

## 2 Materials and methods

### 2.1 Murine experiments

#### 2.1.1 Establishment of the BV mouse model

Eighteen female C57BL/6J mice (age: 3–4 weeks; weight: 16–20 g; supplier: Guangdong Vital River Laboratory Animal Technology Co., Ltd.) were randomly divided into two groups: control (*n* = 9) and bacterial vaginosis (*n* = 9). To induce a persistent estrus state that keratinizes the vaginal epithelium and facilitates *Gardnerella vaginalis* colonization, all mice received daily intraperitoneal injections of 0.005% estradiol benzoate (100 μL) for four consecutive days, starting 3 days before and including the day of inoculation.

Three days after estrogen treatment, vaginal inoculation was conducted. Mice in the BV group were intravaginally inoculated with 20 μL of a suspension of the reference strain *Gardnerella vaginalis* ATCC 49145 (platform No. bio-72726; Beijing Biobw Biotechnology Co., Ltd., Beijing, China) at 1 McFarland standard. Control mice received 20 μL of sterile saline solution. The inoculum was slowly delivered into the deep vagina using a pipette tip until resistance was encountered, and the pipette tip was slowly withdrawn during delivery. Mice were then positioned head-down with an elevated pelvis for 3 min to reduce leakage. This procedure was repeated daily for 6 days.

**Figure 1:**
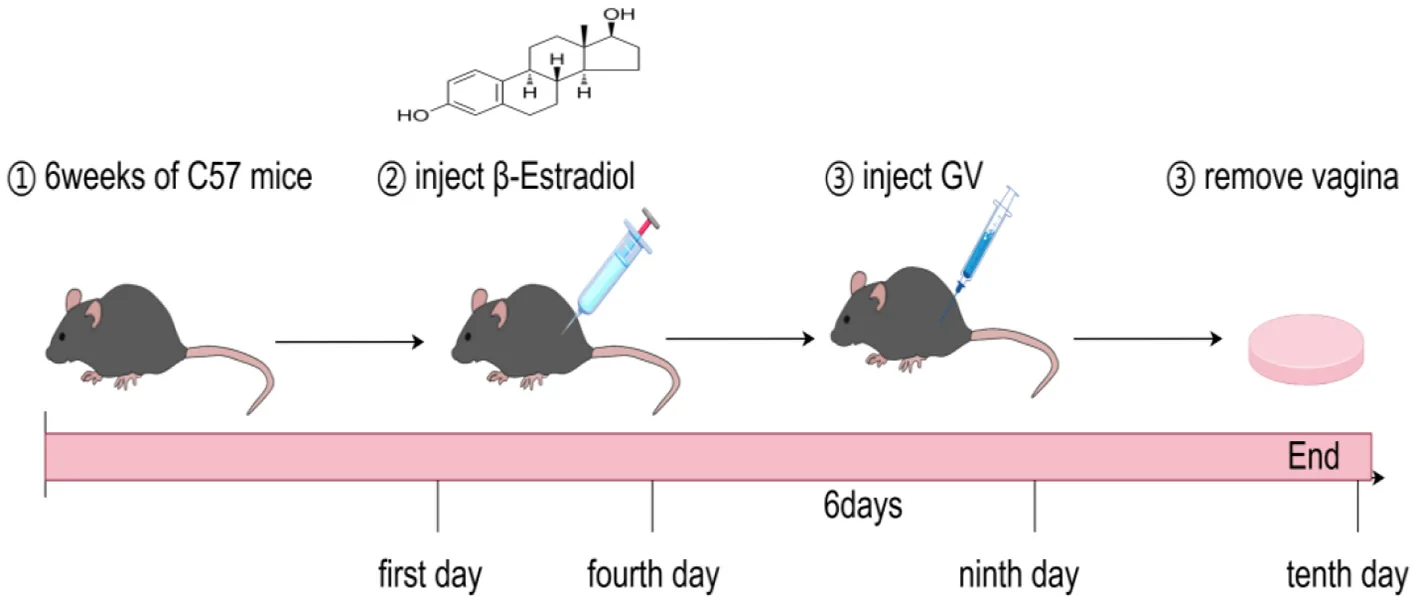
Schematic diagram of the BV mouse model establishment. Mice were pretreated with estradiol benzoate (100 μL of 0.005%, i.p.) for 3 days before and on the day of inoculation. From day 4, mice were inoculated intravaginally daily for 6 days with either *Gardnerella vaginalis* (BV group, 100 μL of 1 MF) or sterile saline (Control group, 100 μL). Created with BioRender.com (https://BioRender.com).

Every 2 days during the modeling phase, food intake, activity levels, and vaginal opening appearance were recorded. On the seventh day after modeling, vaginal lavage fluid was collected by instilling 20 μL of sterile saline solution into the deep vagina, holding it for 2–3 s, and then aspirating it. This process was repeated five times to obtain 100 μL of lavage fluid per mouse. After collection of the vaginal lavage fluid, all mice were euthanized by intraperitoneal (i.p.) injection of an overdose of sodium pentobarbital (150 mg/kg body weight) and dissected for parameter analysis (Figure 1). All procedures complied with the AVMA Guidelines for the Euthanasia of Animals (2020 Edition) and were approved by the Ethics Committee of Fujian Maternity and Child Health Hospital (IACUC-FMCHH-2024-136).

#### 2.1.2 Hematoxylin and Eosin (H&E) staining of mouse vaginal tissues

Vaginal tissue from each mouse was removed after establishing the model. Some samples were snap-frozen and stored at −80 °C, while the remainder were rinsed, dried, and fixed in 4% paraformaldehyde for 24 h. After fixation, tissues underwent standard histological processing, including dehydration, clearing, and paraffin embedding. Serial sections (4–5 μm) were cut, deparaffinized, rehydrated, and stained with hematoxylin and eosin. The stained sections were then dehydrated, cleared, and permanently mounted with neutral balsam.

#### 2.1.3 Western blot analysis

Vaginal tissue samples were ground in liquid nitrogen and placed in pre-chilled microcentrifuge tubes. Cold RIPA lysis buffer (Beyotime, China) containing protease inhibitors (Roche, Germany) was added at a ratio of 1 mL per 100 mg tissue, and the samples were lysed on ice for 30 min with occasional vortexing. After centrifugation at 10,000 rpm for 5 min at 4 °C, the supernatants were collected, and protein concentrations were measured using the BCA assay (Thermo Fisher Scientific, USA).

Equal amounts of protein (20–40 μg) were separated by SDS–PAGE (10% gel) and transferred to PVDF membranes (Millipore, USA). Membranes were blocked with 5% BSA in TBST for 1 h at room temperature and then incubated overnight at 4 °C with primary antibodies as follows: IL-1β (Rabbit, Wuhan Sanying, 26048-1-AP, 1:1,000), IL-10 (Rabbit, Wuhan Sanying, 82191-3-RR, 1:1,000), and β-actin (Rabbit, Wuhan Sanying, 20536-1-AP, 1:5,000). After three washes with TBST, membranes were incubated with HRP-conjugated secondary antibodies (Goat anti-Rabbit or Goat anti-Mouse, FURS I, SA00001-2, 1:10,000) for 1 h at room temperature. Protein bands were visualized using an ECL substrate (Thermo Fisher Scientific, USA) and captured with a chemiluminescence imaging system. β-actin was used as the loading control.

#### 2.1.4 COX-2 fluorescent probe detection in mouse vaginal lavage fluid

The COX-2 fluorescent probe (compound 2a1) was dissolved in dimethyl sulfoxide (DMSO) to prepare a 1 mg/mL stock solution. Immediately before the assay, the stock solution was diluted 100-fold in phosphate-buffered saline (PBS, pH 7.4) to obtain the working solution. For detection, 20 μL of the COX-2 fluorescent probe working solution, 20 μL of mouse vaginal lavage fluid sample, and 160 μL of PBS were sequentially added to each well of a 96-well black opaque microplate. The mixture was gently homogenized, and the microplate was loaded into a multimode microplate reader. Fluorescence intensity was measured using an excitation wavelength of 480 nm and an emission wavelength of 655 nm, according to the previously reported photophysical characterization of compound 2a1 (Xie et al., 2021). Relative fluorescence units were recorded using Gen5 software. Each sample was assayed in triplicate, and blank control wells containing only the probe working solution and PBS were included.

### 2.2 Clinical study

#### 2.2.1 Study population

The study involved 45 women visiting the Gynecology Outpatient Clinic at Fujian Maternal and Child Health Hospital from January to February 2025, either suspected of having BV or undergoing routine vaginal microenvironment checks. This study was approved by the Ethics Committee of Fujian Maternity and Child Health Hospital (Approval No. 2025KY015). All participants provided written informed consent.

Inclusion criteria: (1) History of sexual activity; (2) Presence or absence of vaginitis symptoms.

Exclusion criteria: (1) Concurrent cancer; (2) Active vaginal bleeding or reproductive tract lesions; (3) History of autoimmune disease; (4) Mixed vaginal infections; (5) Systemic or topical antibiotic use within the past 4 weeks; (6) Sexual intercourse within 72 h before sample collection; (7) Known sexually transmitted infections; (8) Vaginal douching, topical medication, or tub bathing within 72 h before sample collection; (9) Recent hysteroscopic or laparoscopic surgery within the past 3 months; (10) Diabetes or major organ dysfunction; (11) Pregnancy, lactation, or menstruation; (12) Incomplete clinical data; (13) Ongoing follow-up or treatment for recurrent vaginitis within the past 3 months.

Group allocation: Participants were classified as BV or non-BV based on Nugent score: the normal microbiota group (normal or reduced-function vaginal flora) and the BV group (diagnosed with BV).

#### 2.2.2 Vaginal microenvironment analysis

Participants were placed in the lithotomy position, and a sterile speculum lubricated with sterile normal saline was gently inserted to expose the vaginal wall. Vaginal secretions were collected from the upper lateral vaginal wall using a sterile cotton swab, avoiding contact with the cervix and external genitalia. The swab was rotated gently against the vaginal wall to ensure adequate sample collection and was then immediately transferred into a sterile tube containing 1 mL of sterile normal saline for elution. All samples were collected by trained clinical personnel following a standardized vaginal secretion collection protocol. The supernatant was collected and stored at −80 °C for no longer than 2 weeks before COX-2 fluorescence analysis.

#### 2.2.3 COX-2 fluorescent probe detection in human vaginal secretions

The COX-2 fluorescent probe (compound 2a1) was dissolved in DMSO to prepare a 1 mg/mL stock solution. Immediately before the assay, the stock solution was diluted 100-fold in PBS to obtain the working solution. For detection, 20 μL of the COX-2 fluorescent probe working solution, 20 μL of human vaginal secretion supernatant, and 160 μL of PBS were sequentially added to each well of a 96-well black microplate. After mixing, the microplate was loaded into a multimode reader to measure fluorescence at an excitation wavelength of 480 nm and an emission wavelength of 655 nm. Fluorescence was recorded with Gen5 software, and each sample was tested in triplicate. Blank control wells were also included.

The operator performing the fluorescence assay was blinded to the Nugent score, vaginal microecological results, and clinical group until all fluorescence measurements had been completed.

### 2.3 Statistical analysis

Statistical analyses were conducted using SPSS (version 26.0). All continuous variables were assessed for normality using the Shapiro-Wilk test. Normally distributed variables were compared using independent-samples *t*-test or ANOVA with Tukey's *post-hoc* test; non-normal variables were compared using Mann–Whitney *U* or Kruskal–Wallis tests. Categorical variables were analyzed using chi-square or Fisher's exact test as appropriate. *P* < 0.05 was considered statistically significant.

## 3 Results

### 3.1 Mouse experimental section

#### 3.1.1 Vulvar condition in mice

Both groups of mice underwent a 4-day estrogen priming regimen. On the fourth day, differential treatments were administered according to group allocation: mice in the BV model group received intravaginal perfusion of a *Gardnerella vaginalis* suspension, while control mice received sterile saline. This treatment continued for 6 days. After model establishment, systematic observation and evaluation of the mouse vaginas were conducted. Mice in the BV model group exhibited typical signs of vaginal inflammation, characterized by marked redness, swelling, mucosal congestion, and turbid vaginal lavage fluid, indicating abnormal secretions or inflammatory exudates. In contrast, control mice showed a normal vaginal appearance without redness, swelling, or mucosal congestion, and their vaginal lavage fluid was clear and transparent.

#### 3.1.2 Histopathological examination of vaginal tissues via H&E staining

**Figure 2:**
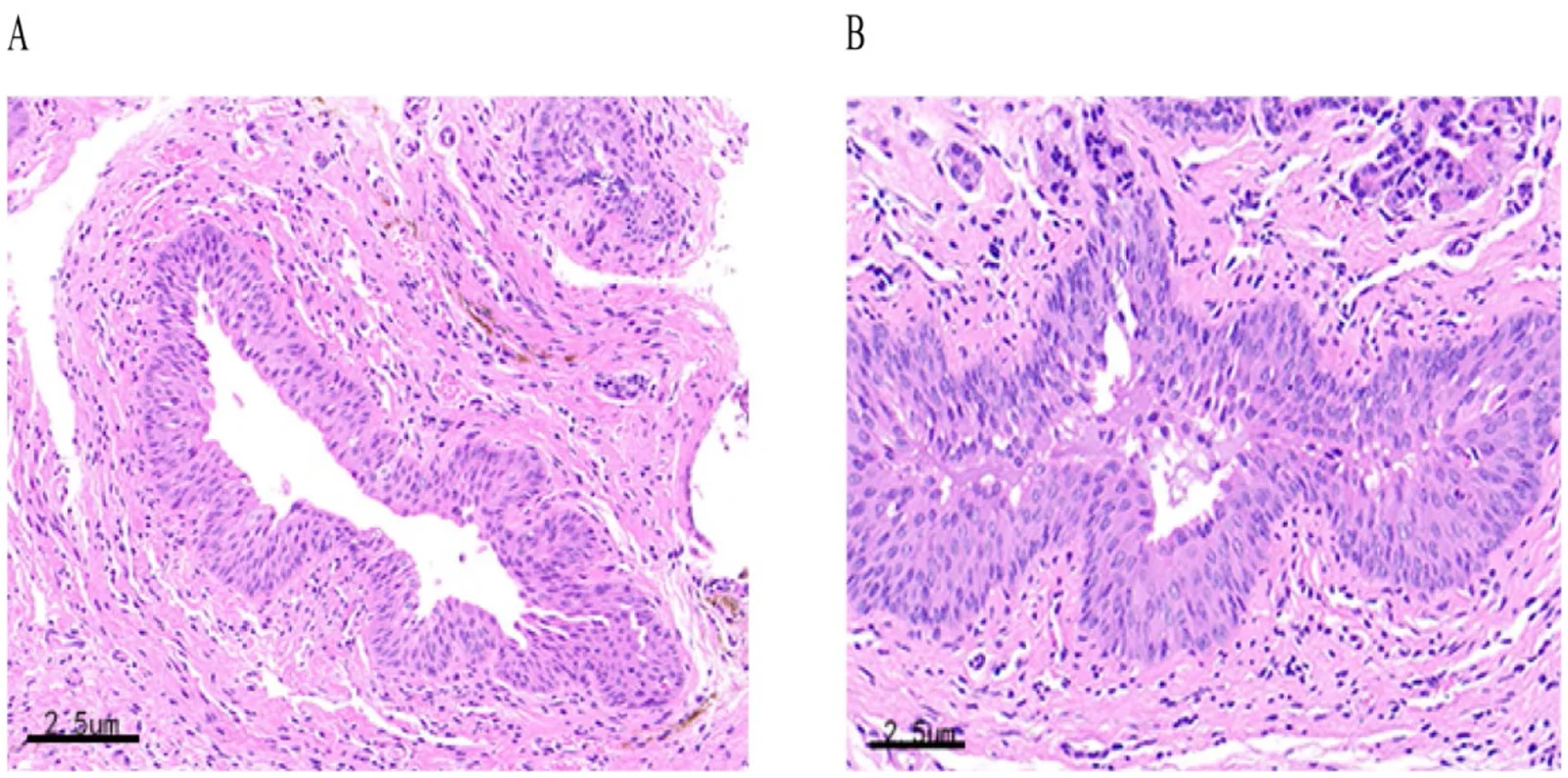
H&E-stained images of vaginal tissues from different mouse groups. **(A)** H&E-stained vaginal tissue from the control group. **(B)** H&E-stained vaginal tissue from the bacterial vaginosis group.

After euthanasia, vaginal tissues were dehydrated, embedded in paraffin, sectioned, and stained with H&E. Histological analysis showed that control mice had intact vaginal epithelium without signs of inflammation, whereas BV group mice exhibited epithelial shedding and significant inflammatory cell infiltration (Figure 2).

#### 3.1.3 Protein expression analysis by western blotting

**Figure 3:**
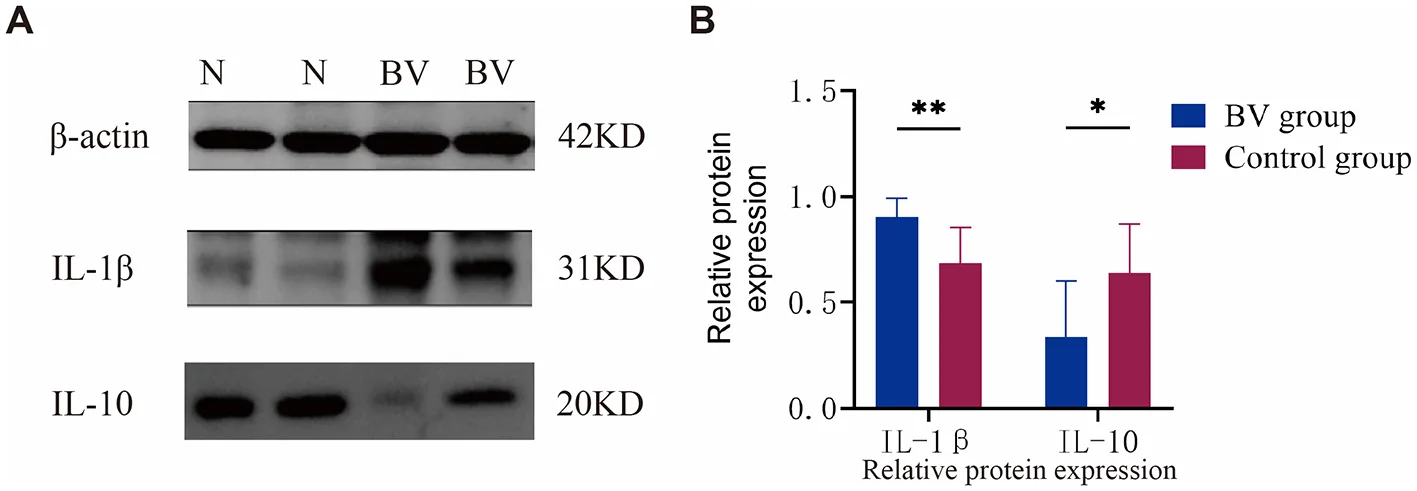
Panel A represents the representative western blot bands, and panel B represents the quantitative analysis of IL-1β and IL-10 protein expression. All data are expressed as mean ± SD. *P < 0.05; **P < 0.01.

Western blot analysis showed that interleukin-1β (IL-1β) levels were significantly increased and IL-10 levels were significantly decreased (*P* < 0.05) in the BV group compared with the control group in mouse vaginal tissues (Figure 3).

#### 3.1.4 Detection of COX-2 expression in mouse vaginal lavage fluid

**Figure 4:**
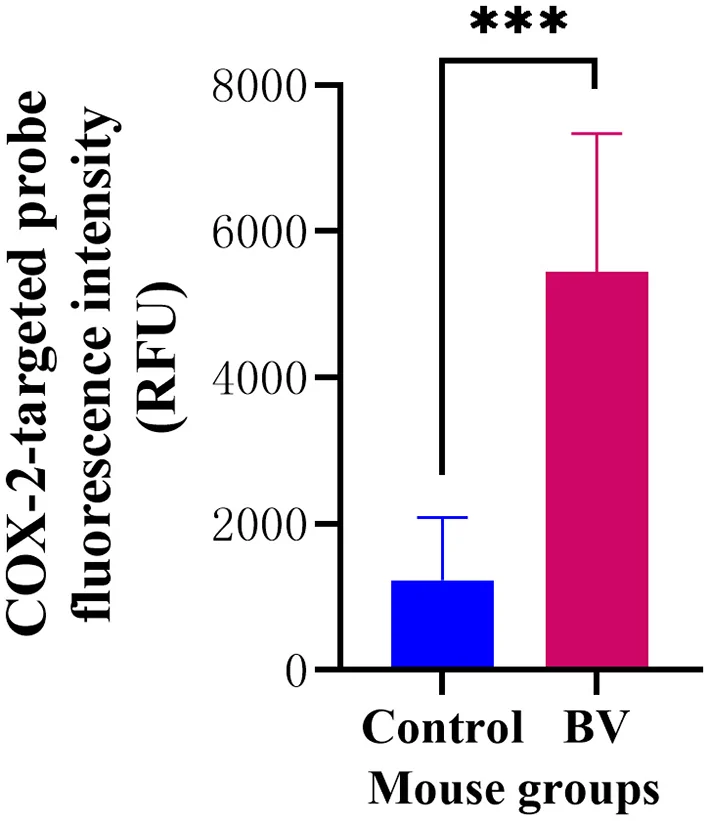
COX-2 fluorescent probe detection results for vaginal lavage fluid in control and BV group mice. All data are expressed as mean ± SD. ***P < 0.001.

Vaginal lavage fluid was collected from both control and BV group mice after model establishment. COX-2 expression was measured with a fluorescent probe. The BV group exhibited a significantly higher fluorescence signal compared with the control group (*P* < 0.05; Figure 4).

### 3.2 Human study results

#### 3.2.1 Baseline characteristics of the study population

Table 1 presents the baseline clinical characteristics and vaginal microecological test results for the 45 women included in the study. The median age was 36 years (range: 19–61; IQR: 30–45). Twenty-five women (55.56%) were asymptomatic and underwent testing for routine diagnostics. Reported symptoms included vulvar pruritus (33.33%), increased vaginal discharge (17.78%), abnormal discharge (8.89%), and malodorous discharge (13.33%). Some women experienced multiple symptoms simultaneously. Vaginal cleanliness grades were as follows: Grade I (6.67%), Grade II (37.78%), Grade III (46.67%), and Grade IV (8.89%).

**Table 1:** Baseline characteristics of the study population.

Variable	Cases	Median (interquartile range)/ composition ratio (%)
Age (years)	45	36 (30–45)
Symptoms	45	
Asymptomatic	25	55.56 (25/45)
Vulvar pruritus	15	33.33 (15/45)
Abnormal discharge	4	8.89 (4/45)
Increased vaginal discharge	8	17.78 (8/45)
Malodorous discharge	6	13.33 (6/45)
Vaginal cleanliness grades	45	
Grade I	3	6.67 (3/45)
Grade II	17	37.78 (17/45)
Grade III	21	46.67 (21/45)
Grade IV	4	8.89 (4/45)
Vaginal microecology status	45	
Normal microflora	6	13.33 (6/45)
Vaginal dysbiosis	39	86.67 (39/45)
Decreased function	19	48.72 (19/39)
BV	20	51.28 (20/39)

Values are expressed as median (interquartile range) or percentage composition.

Vaginal microecological testing showed that six women (13.33%) had normal microflora, while 39 women (86.67%) had vaginal dysbiosis. Of the dysbiosis cases, 19 (48.72%) had normal flora with decreased function, and 20 (51.28%) were diagnosed with BV.

#### 3.2.2 Comparison of COX-2 fluorescent probe detection values in different populations

**Figure 5:**
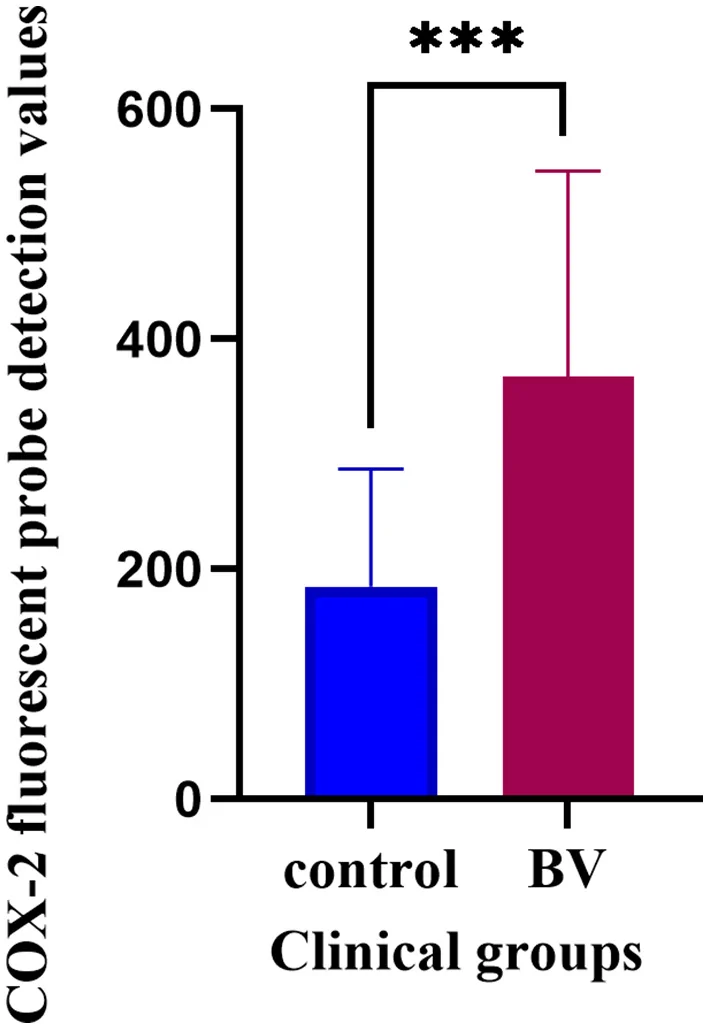
COX-2 fluorescent probe detection levels across different group. Data are presented as mean ± SD. *P < 0.05, **P < 0.01, ***p < 0.001.

In the animal experiment module, COX-2 fluorescent probe detection values in the vaginal lavage fluid of mice with BV were significantly higher than those in the control group. Previous literature has confirmed that COX-2 gene expression is elevated in a mouse model of BV induced by *Gardnerella vaginalis*. In the present study, COX-2 detection values were compared between normal women and those with BV. The mean ± SD values were 184.76 ± 102.38 in the normal group and 367.85 ± 178.16 in the BV group, with a statistically significant difference (*t* = −4.33, *P* < 0.05). This indicates that COX-2 expression was significantly lower in the normal group compared with the BV group (Table 2), after the description of COX-2 fluorescence levels in clinical samples Figure 5.

**Table 2:** Differential analysis of COX-2 fluorescent probe detection values between the control group and the BV group.

Group	Cases (*n*)	Mean ±SD	*t*-value	*P*-value
Control group	25	184.76 ± 102.38	−4.33	<0.001
BV group	20	367.85 ± 178.16		

Vaginal cleanliness is an important parameter in the diagnosis of vaginitis, with abnormal cleanliness indicating the presence of white blood or pus cells. In this study, COX-2 fluorescent probes were used to assess vaginal secretions, and detection values were compared across cleanliness grades. The COX-2 values were 67.67 in grade I, 218.59 in grade II, 262.43 in grade III, and 636.50 in grade IV. Significant differences were observed between grades I and II, III, and IV; grade II and I, IV; and grade III and I, IV (*P* < 0.05; Table 3). Because of the small sample sizes in grades I and IV, and considering that clinical practice often groups grades I–II as non-vaginitis and grades III–IV as vaginitis, the study reclassified the groups accordingly. Analysis showed that COX-2 detection values were significantly lower in grades I–II (195.95 ± 106.23) compared with grades III–IV (322.28 ± 186.57; *P* < 0.05; Table 4).

**Table 3:** Differential analysis of COX-2 fluorescent probe detection values based on vaginal cleanliness grades.

Cleanliness grades	Cases (*n*)	Mean ±SD	*F*-value	*P*-value
Grades I	3	67.67 ± 10.07	18.49	<0.001
Grades II	17	218.59 ± 98.78		
Grades III	21	262.43 ± 129.19		
Grades IV	4	636.50 ± 102.39		

**Table 4:** Differential analysis of COX-2 fluorescent probe detection values by grouped vaginal cleanliness grades.

Cleanliness grades	Cases (*n*)	Mean ±SD	*t*-value	*P*-value
Grades I–II	20	195.95 ± 106.23	−2.70	0.010
Grades III–IV	25	322.28 ± 186.57		

#### 3.2.3 Comparison of dominant vaginal microbiota with COX-2 fluorescent probe detection values

*Lactobacillus* species are the predominant flora in a healthy vaginal environment, whereas bacterial vaginosis is characterized by the presence of Gram-negative small bacilli or coccobacilli. This study compared dominant microbial groups with COX-2 fluorescent probe detection values. Twenty-five participants had Gram-positive large bacilli, with a mean ± SD COX-2 value of 184.76 ± 102.38, whereas 20 participants had Gram-negative small bacilli or coccobacilli, with a higher mean ± SD COX-2 value of 367.85 ± 178.16. The *t*-value of −4.33 indicated a statistically significant difference between the two groups (*P* < 0.05). COX-2 fluorescent probe detection values were significantly lower in women with predominantly Gram-positive bacilli compared with those with predominantly Gram-negative bacilli or coccobacilli (*P* < 0.05; Table 5).

**Table 5:** Differential analysis of COX-2 fluorescent probe detection values based on dominant vaginal flora.

Dominant flora	Cases (*n*)	Mean ±SD	*t*-value	*P*-value
Gram-positive bacilli	25	184.76 ± 102.38	−4.33	<0.001
Gram-negative bacilli or coccobacilli	20	367.85 ± 178.16		

#### 3.2.4 Comparison of Nugent score with COX-2 fluorescent probe detection values

**Figure 6:**
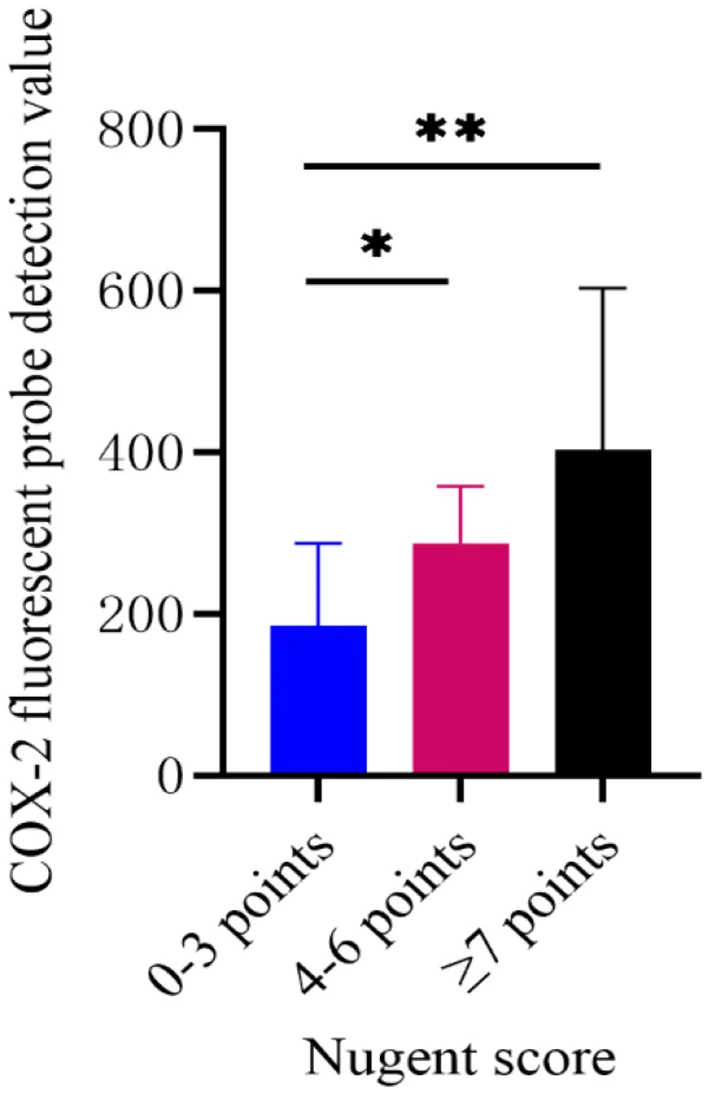
COX-2 fluorescent probe detection levels across different Nugent score groups. Data are presented as mean ± SD. *P < 0.05, **P < 0.01.

The Nugent score, a key diagnostic tool for BV, is determined by examining Gram-stained vaginal swabs. Scores range from 0–3 (normal), 4–6 (intermediate), and ≥7 (BV). In this study, COX-2 fluorescent probe detection values were compared across Nugent score categories. Twenty-five women with scores of 0–3 had a mean ± SD COX-2 value of 184.76, six women with scores of 4–6 had a mean ± SD of 287.71, and 14 women with scores ≥7 had a mean ± SD of 402.43. Statistical analysis showed a significant difference in COX-2 levels among Nugent score groups (*F* = 11.26, *P* < 0.05; Table 6). A bar chart indicated that COX-2 levels increased with higher Nugent scores. *Post hoc* analysis confirmed that COX-2 levels in the 0–3 group were significantly lower than those in the 4–6 and ≥7 groups (Figure 6).

**Table 6:** Differential analysis of COX-2 fluorescent probe detection values based on Nugent score.

Nugent score group	Cases (*n*)	Mean ±SD	*F*	*P*
0–3 points	25	184.76 ± 102.38	11.26	<0.001
4–6 points	6	287.71 ± 70.32		
≥7 points	14	402.43 ± 200.50		

#### 3.2.5 Comparison of clue cell density with COX-2 fluorescent probe detection values

**Figure 7:**
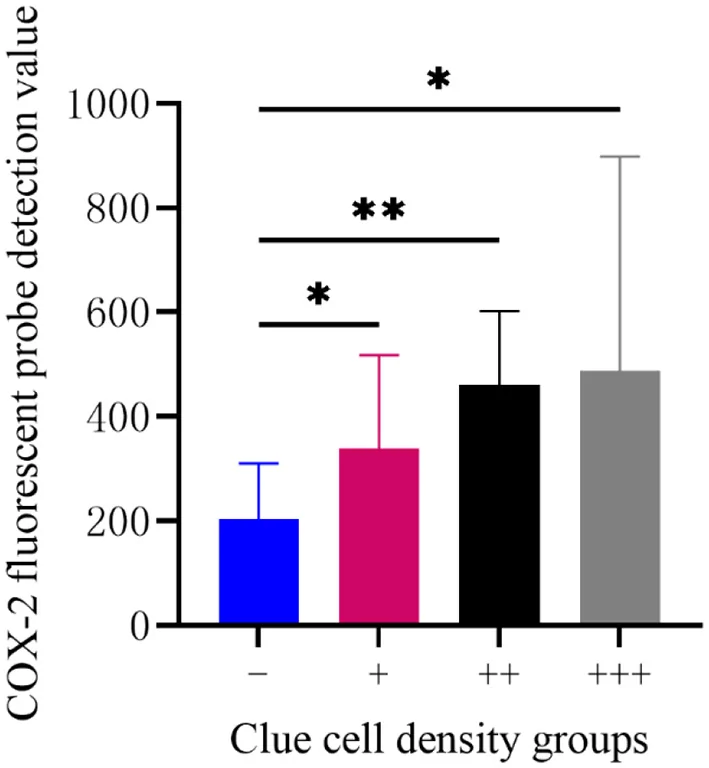
Relationship between clue cell density and COX-2 fluorescent probe detection values. Data are presented as mean ± SD. *P < 0.05, **P < 0.01.

Clue cells are an essential diagnostic marker for BV according to Amsel's criteria. This study analyzed the relationship between clue cell density and COX-2 fluorescent probe detection values. The results demonstrated a progressive increase in COX-2 values with higher clue cell densities: 203.67 ± 106.51 for 30 participants without clue cells, 339.00 ± 178.85 for nine participants with “+,” 460.00 ± 141.78 for four participants with “++,” and 487.50 ± 410.83 for two participants with “+++.” Significant differences in COX-2 values were observed among the groups (*F* = 6.94, *P* < 0.05; Table 7; Figure 7). Moreover, the clue cell–negative group had significantly lower COX-2 values compared with all other groups.

**Table 7:** Differential analysis of COX-2 fluorescent probe detection based on clue cell density.

Clue cell density group	Cases	Mean ±SD	*F*	*P*
Negative (–)	30	203.67 ± 106.51	6.94	0.00
+	9	339.00 ± 178.85		
++	4	460.00 ± 141.78		
+++	2	487.50 ± 410.83		

#### 3.2.6 Comparison of microbial density in vaginal microbiota and COX-2 fluorescent probe detection values

**Figure 8:**
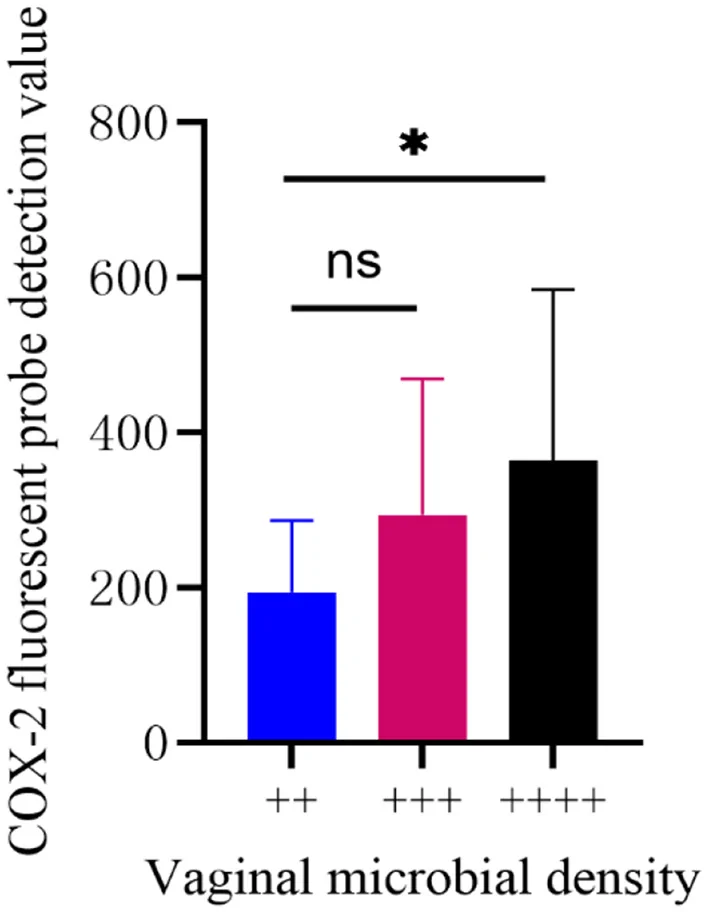
Correlation between vaginal microbiota density and COX-2 fluorescent probe detection values. Data are presented as mean ± SD. *P < 0.05; ns indicates no significant difference.

This study examined the relationship between vaginal microbial density and COX-2 fluorescent probe detection values. In 18 women, an average bacterial count of 10–99 per field under oil immersion corresponded to a COX-2 value of 193.61 ± 92.79. For 19 women with counts of ≥100 per field, the COX-2 value was 293.58 ± 175.70. In eight women, bacteria densely covering cells resulted in a COX-2 value of 364.13 ± 220.18. Significant differences in COX-2 values were found among groups with varying microbial densities (*F* = 3.75, *P* < 0.05), with values in the 10–99 group significantly lower than those in the “dense coverage” group (*P* < 0.05; Table 8). An upward trend in mean COX-2 values was observed across groups (Figure 8).

**Table 8:** Differential analysis of COX-2 fluorescent probe detection based on vaginal microbial density.

Vaginal microbial density	Cases	Mean ±SD	*F*	*P*
10–99 per field	18	193.61 ± 92.79	3.75	0.03
≥100 per field	19	293.58 ± 175.70		
Dense coverage	8	364.13 ± 220.18		

## 4 Discussion

BV is a common infection among women of reproductive age, caused by an imbalance in the vaginal microbiota. Current BV diagnostic modalities have notable limitations: Amsel's criteria are subjective and time-consuming, Nugent scoring is complex and labor-intensive, and quantitative inaccuracies hinder objective severity assessment. Molecular diagnostics provide high sensitivity and specificity; however, their high cost limits their availability for routine, immediate testing, confining their primary use to research settings. This study aimed to address these unmet clinical needs by evaluating a novel COX-2-targeting fluorescent probe as an adjunctive tool for quantitative assessment of BV-related inflammatory severity. COX-2 is a key enzyme involved in inflammation, catalyzing the conversion of arachidonic acid to prostaglandins (Lee et al., 2024). Its expression is markedly upregulated in a mouse model of BV induced by *Gardnerella vaginalis* (Kwon et al., 2024; Li et al., 2023; Joo et al., 2011). Given these characteristics, applying COX-2 probes may offer an adjunctive quantitative readout of BV-associated inflammatory severity for monitoring therapeutic efficacy. The present study employed a novel COX-2 fluorescent probe developed by the Fujian Institute of Microbiology (Xie et al., 2021). This probe features simplified synthesis and reduces the impact of molecular weight increases on its physicochemical properties.

In this study, a murine model of BV was successfully established through vaginal inoculation with pathogenic bacteria in combination with estrogen pretreatment. This method promoted colonization of GV within the murine vaginal environment, induced dysbiosis of the vaginal microbiota, and subsequently triggered bacterial vaginal inflammation. Morphological examination of vaginal tissues revealed that mice in the BV model group exhibited pronounced vaginal edema, congestion, and turbid vaginal lavage fluid compared with the control group. Histopathological evaluation using H&E staining demonstrated epithelial cell desquamation and infiltration of inflammatory cells in the vaginal tissues of BV-affected mice. Although the murine BV model established using *Gardnerella vaginalis* and estrogen pretreatment allows evaluation of COX-2 probe performance, we acknowledge that the murine vaginal microbiota differs substantially from humans, typically lacking Lactobacillus dominance. Therefore, results from this model mainly demonstrate proof-of-concept for the probe rather than fully recapitulating human BV pathophysiology. In addition, model establishment was confirmed by macroscopic signs, histopathology, and cytokine changes, which reflect local inflammation; the vaginal bacterial burden was not directly quantified, which is a further limitation that future studies should address to confirm BV-like colonization and dysbiosis.

In BV, *Lactobacillus* species are replaced by anaerobic bacteria, including GV, *Ureaplasma vaginalis, Ureaplasma urealyticum*, and *Mycoplasma hominis* (Abou Chacra et al., 2021). GV elicits immune responses in the vaginal and cervical regions by activating macrophages, predominantly classical pro-inflammatory macrophages (M1) which are defined by their pro-inflammatory properties, notably the secretion of cytokines such as IL-1β (Shapouri-Moghaddam et al., 2018; Liu et al., 2021). In advanced stages of BV-related inflammation, macrophages undergo a phenotypic transition from the pro-inflammatory M1 phenotype to the anti-inflammatory M2 phenotype, thereby promoting tissue repair (Mosser and Edwards, 2008). M2 macrophages primarily secrete anti-inflammatory cytokines, including interleukin-10 (IL-10; Cortés-Morales et al., 2023). Western blot analysis was performed to assess the expression levels of IL-10 and IL-1β in this study. The results demonstrated a significant increase in the pro-inflammatory cytokine IL-1β and a decrease in the anti-inflammatory cytokine IL-10 in BV-model mice, indicating successful establishment of the BV mouse model. Furthermore, detection of COX-2 in vaginal lavage fluid using a fluorescent probe revealed significantly higher fluorescence values in the BV group compared with the control group (*P* < 0.05). These results were consistent with the findings of Li et al., 2023, supporting the potential utility of COX-2 fluorescent probes for quantifying BV-associated inflammatory activity. However, the limitations of this study should be acknowledged—particularly the differences in vaginal microbiota composition between mice and humans, which may influence the clinical applicability of these findings.

In the human study, a comparative analysis of vaginal microbiota test results and COX-2 fluorescent probe detection values in vaginal secretions was conducted between women with BV and those without. The findings revealed that COX-2 fluorescent probe detection values were significantly higher in the BV group compared with the normal group, with statistical significance. These results align with the outcomes observed in the corresponding murine study.

Vaginal cleanliness grading is an important metric in routine gynecological assessments for evaluating the vaginal microecological environment and inflammatory status. It is primarily determined through microscopic examination of vaginal secretion components. The grading definitions and criteria are based on the proportions of white blood cells, epithelial cells, *Lactobacillus*, and pathogenic microorganisms (such as heterotrophic bacteria, fungi, and *Trichomonas*) present in vaginal secretions. Vaginal cleanliness is categorized into grades I–IV, reflecting the presence of vaginal infection or microbiota dysbiosis. Clinically, grades I–II are generally classified as non-vaginitis, whereas grades III–IV are associated with inflammatory conditions. In this study, COX-2 fluorescent probe detection values were initially compared across the four cleanliness grades. The results demonstrated significant differences among the groups, with mean values increasing alongside cleanliness grade. Further analysis, which reclassified the grades into two categories (grades I–II and grades III–IV), revealed significantly higher COX-2 detection values in the III–IV group.

Participants with Gram-negative bacilli/bacteroides as the predominant vaginal microbiota had significantly higher COX-2 fluorescent probe detection values in vaginal secretions compared with those whose microbiota was dominated by Gram-positive bacilli. Typically, the normal vaginal microbiota in women is dominated by *Lactobacillus*, which appears as Gram-positive bacilli on Gram staining. In contrast, Gram-negative bacilli/bacteroides—primarily comprising *Gardnerella vaginalis, Prevotella*, and *Campylobacter*—are prevalent in patients with BV (Swidsinski et al., 2023). Notably, none of the participants with Gram-positive bacilli as the dominant flora were diagnosed with vaginitis, whereas all participants with Gram-negative bacilli/bacteroides were diagnosed with BV. This finding reinforces the significant difference in COX-2 fluorescent probe detection values between BV and non-BV patients.

The Nugent score is recognized as the gold standard for diagnosing BV (Muzny et al., 2022). It evaluates vaginal microbiota dysbiosis through semi-quantitative analysis of bacterial morphology and enumeration in Gram-stained vaginal smear preparations. This method uses a scoring system based on the morphology and relative abundance of three bacterial types: Gram-positive bacilli, Gram-negative or Gram-variable bacilli, and curved Gram-negative or Gram-variable bacilli. Individuals are categorized into three groups according to the final score: (1) 0–3 points, indicating normal vaginal microbiota dominated by *Lactobacillus*; (2) 4–6 points, indicating intermediate BV (partial microbiota dysbiosis requiring correlation with clinical symptoms); and (3) ≥7 points, confirming BV (characterized by reduced *Lactobacillus* and increased pathogenic bacteria; Nugent et al., 1991). Comparison of COX-2 fluorescent probe detection values across Nugent score groups revealed significant differences. Multiple comparison analyses showed that the 0–3 point group had significantly lower COX-2 values compared with the other two groups. These findings indicate distinct COX-2 levels between women with BV and those without vaginitis, supporting its use for severity stratification among women with BV.

Clue cells are vaginal epithelial cells extensively colonized by bacteria, primarily Gardnerella and anaerobes. These bacteria form granular or punctate structures that give the cells a “peppered” appearance under microscopic examination and cause blurred cell edges. The presence of clue cells is strongly indicative of BV, and a positive clue cell test constitutes one of the critical criteria in the Amsel diagnostic framework for BV. In this study, bar chart analysis demonstrated a positive correlation between increasing clue cell density and higher mean COX-2 fluorescent probe detection values. Multiple comparison analyses further showed that individuals without clue cells had significantly lower COX-2 values than those with clue cells, underscoring its correlation with the degree of BV-associated dysbiosis and inflammation.

The biological rationale for targeting COX-2 in BV warrants explicit consideration. Classical BV is characterized as a polymicrobial dysbiosis accompanied by a comparatively restrained mucosal inflammatory response, lacking the overt leukocyte influx typical of aerobic vaginitis or vulvovaginal candidiasis (Bradshaw et al., 2025). This raises an apparent paradox in proposing an inflammatory enzyme as a readout for a condition of limited inflammation. Two considerations are relevant. First, the “limited” inflammation of BV is defined relative to frankly inflammatory vaginitides and does not denote absence of mucosal immune activation; BV-associated organisms, particularly *G. vaginalis*, induce epithelial and macrophage activation, IL-1β release, and prostaglandin synthesis, as reflected in our murine data (Kwon et al., 2024; Li et al., 2023; Joo et al., 2011; Gilbert et al., 2025). Second, the host inflammatory response in BV is markedly heterogeneous between individuals, and it is this graded, often subclinical inflammatory activity—rather than dysbiosis *per se*—that has been linked to preterm birth, pelvic inflammatory disease, and recurrence (Bradshaw et al., 2025; Vodstrcil et al., 2025). A quantitative inflammatory readout is therefore complementary to, rather than redundant with, Nugent scoring, which enumerates bacterial morphotypes and is by design blind to host response. On this basis we position the COX-2 probe not as a diagnostic test for BV, but as a measure of inflammatory activity applied *after* BV has been established by standard microbiological criteria.

The principal limitation of this study concerns specificity. COX-2 is a broadly inducible enzyme, and our design compared women with BV against women with normal vaginal microbiota only; no disease-control groups—such as vulvovaginal candidiasis, aerobic vaginitis, cervicitis, or *Trichomonas* vaginitis—were included. The objective here was to quantify the severity of the host inflammatory response within established BV—the dimension that Amsel's and Nugent's binary criteria leave ungraded—rather than to discriminate BV from other vaginitides. Consequently, the present data cannot determine whether the elevated fluorescence signal reflects BV-specific pathophysiology or simply the presence of lower genital tract inflammation, and we acknowledge that the probe may primarily report inflammatory activity rather than BV-specific processes. The correlations observed with Nugent score, clue cell density, and bacterial load are compatible with either interpretation, since inflammatory intensity and the degree of dysbiosis co-vary (Gilbert et al., 2025). We therefore make no claim of diagnostic specificity for BV. Determining whether the probe carries discriminative information beyond generic inflammation will require a prospective study incorporating the disease-control groups named above, which is the principal direction of our ongoing work. Nevertheless, within a standardized clinical and microbiological workflow, the probe still provides an objective, quantitative index of BV-associated inflammatory severity that may inform individualized treatment decisions. Besides, the sample size was relatively small, comprising 20 BV cases and 25 control subjects. This limitation primarily resulted from the 2-month project duration and the need for consistent sample collection by a single deputy chief gynecologist. Additionally, the use of stringent inclusion and exclusion criteria, aimed at ensuring the reliability of results, further constrained the sample size. Notably, previous studies investigating the role of COX-2 in disease diagnosis have also used small sample sizes, such as one involving 40 participants (Kumari et al., 2021). Although direct evidence linking COX-2 to the pathogenesis of BV is limited, studies using BV mouse models infected with *Gardnerella vaginalis* have demonstrated significantly increased COX-2 expression (Kwon et al., 2024; Li et al., 2023; Joo et al., 2011). These findings are consistent with our observations in both murine and human subjects. Despite these limitations, this study represents the first exploratory application of COX-2 fluorescent probes for the adjunctive quantitative assessment of BV-associated lesion severity, providing preliminary evidence that may guide future large-scale investigations. Furthermore, the absence of a standardized detection threshold for COX-2 levels in BV currently limits its further application in severity stratification and clinical decision-making. Notably, this study did not evaluate conventional diagnostic-performance metrics—such as ROC analysis, sensitivity, specificity, predictive values, or optimal fluorescence cut-off thresholds—because the probe is positioned as an adjunctive quantitative biomarker rather than a stand-alone diagnostic replacement, and the current single-center cohort with limited grouping would yield unstable estimates. Formal diagnostic-accuracy analyses are planned as a core endpoint of these future multicentre studies. Therefore, multicenter studies with larger sample sizes are needed to validate the generalizability of these findings.

In summary, the COX-2 fluorescent probe assay shows promise as an adjunctive severity-assessment tool. By evaluating detection values, this method may help assess the severity of BV lesions. Compared with traditional BV detection techniques, it reduces human factor interference to some extent and can assist in determining disease severity. Accordingly, this approach has potential to improve clinical BV treatment and reduce the incidence of missed diagnoses.

## 5 Conclusion

The findings of this study indicate that COX-2 fluorescent probe detection values are significantly elevated in mouse models of BV, supporting its utility for quantifying BV-associated inflammatory severity. In human studies, COX-2 fluorescent probe values differed substantially between BV patients and healthy controls, with significantly higher levels in the BV cohort. Comparative analyses with BV-associated vaginal microbiota indices demonstrated significant variability in COX-2 values across different diagnostic subgroups. Histogram analysis further revealed an upward trend in mean COX-2 values correlating with increased disease severity. Notably, individuals without clue cells exhibited significantly lower COX-2 values than those with clue cell positivity, and participants with Nugent scores of 0–3 had significantly lower values than those in other diagnostic groups.

These results suggest that COX-2 fluorescent probe values could serve as a marker for assessing BV severity. This capability may help clinicians select appropriate treatments, evaluate prognoses, and conduct prevalence surveys of vaginal infections, thereby enhancing the comprehensiveness of lower genital tract secretion analyses. With validation using laboratory and clinical specimens, the COX-2 fluorescent probe offers advantages such as simplicity, rapidity, ease of interpretation, reduced human error, and low synthesis costs. Future research should involve multicenter studies with larger sample sizes to confirm the generalizability of these findings and to establish quantitative relationships, facilitating the clinical application of this probe.

## Data Availability

The raw data supporting the conclusions of this article will be made available by the authors, without undue reservation.
